# Diseases of the Nose and Throat

**Published:** 1911-12

**Authors:** 


**Diseases of the Nose and Throat.** By St. Clair Thomson, M.D., F.R.C.P., F.R.C.S. Pp. xvi, 791. London: Cassell and Co. 1911.

Rhino-laryngology has become a large territory, and any text-book which covers the whole ground and aims at a clear enunciation of the general principles and practice of this speciality must perforce reach the dimensions of a goodly-sized volume.

The work is divided into eleven parts, Part I dealing with embryology and physiology, methods of examination and general symptoms, treatment and the dangers of operations. We find that a very practical and clear description of bronchoscopy and œsophagoscopy is included in the section on methods of examination. A very useful topic is the cleansing of the nose, but we should have liked to see a warning note on the dangers that frequently attend the indiscriminate use of the nasal douche.

Part II consists of diseases of the nose. The special chapter on "taking cold, infectious catarrh" will be appreciated by all practitioners, but the space devoted to injuries to the nose and resulting deformities might with advantage have been more liberal.

Diseases of the accessory sinuses is treated in a masterly way, but would have been more complete with fuller reference to the different operations which are employed in the treatment of these affections.

In addition to a very large number of excellent illustrations in colour and in black and white, the author has employed diagrams to elucidate the text, and these are in many instances most helpful, such as that of a horizontal section of the pharynx to show the anatomical relations of the tonsils and the retro-pharyngeal glands.

In Part IV diseases of the naso-pharynx, diseases of the pharynx and tonsils, the larynx, trachea and œsophagus are followed by a section on tuberculosis and syphilis of the upper air passages and other infective diseases, and we consider that the author's plan of dealing simultaneously with these affections in the naso-pharynx and larynx has much to recommend it. Sections on acute specific fevers in the nose and throat, and the nose and throat in some general affections, and one on some special operations, lead one to the final pages, in which the author gives a number of very useful formulæ.

Professor St. Clair Thomson's treatise on the nose and throat is clearly written, well printed and richly illustrated, and is certain to take a leading place as one of the most authoritative, complete and scientific that has ever appeared.

